# How the Probabilistic Structure of Grammatical Context Shapes Speech

**DOI:** 10.3390/e22010090

**Published:** 2020-01-11

**Authors:** Maja Linke, Michael Ramscar

**Affiliations:** Department of Linguistics, University of Tuebingen, Wilhelmstraße 19, 72074 Tuebingen, Germany; michael.ramscar@uni-tuebingen.com

**Keywords:** speech variance, communicative efficiency, sampling invariance, power laws, communicative distributions

## Abstract

Does systematic covariation in the usage patterns of forms shape the sublexical variance observed in conversational speech? We address this question in terms of a recently proposed discriminative theory of human communication that argues that the distribution of events in communicative contexts should maintain mutual predictability between language users, present evidence that the distributions of words in the empirical contexts in which they are learned and used are geometric, and thus support this. Here, we extend this analysis to a corpus of conversational English, showing that the distribution of grammatical regularities and the sub-distributions of tokens discriminated by them are also geometric. Further analyses reveal a range of structural differences in the distribution of types in parts of speech categories that further support the suggestion that linguistic distributions (and codes) are subcategorized by context at multiple levels of abstraction. Finally, a series of analyses of the variation in spoken language reveals that quantifiable differences in the structure of lexical subcategories appears in turn to systematically shape sublexical variation in speech signal.

## 1. Introduction

The words produced in conversational speech often differ substantially from the acoustic signals supposed by canonical dictionary forms [1,2]. The extent to which articulated forms deviate from dictionary models correlates with average word frequency, such that there is a general tendency for shorter and faster articulation in more probable words. This property of speech codes is often taken to suggest that human speech is shaped by the competing requirements of maximizing the success of message transmission while minimizing production effort in ways similar to those described by information coding solutions for electronic message transmission. There are, however, some critical differences between speech and the communication model described by information theory [3]: whereas information theory is concerned with defining the properties of variable length codes optimized for efficient communication in discrete, memoryless systems, human communication codes, at first blush at least, appear neither systematic [3] nor systematically discrete [2,4] or memoryless [5].

In regard to the first point, systematicity, humans learn to communicate by the gradual discrimination of functional (task-relevant) speech dimensions from the samples to which they are exposed, yet because lexical diversity in language samples increases nonlinearly over space and time, the divergence between the samples individuals are exposed to increases as their experience of the linguistic environment grows [5]. A system defined by a probabilistic structure would appear to require that events be distributed in a way that allows the relationships between events probabilities to remain stable independent of the sample size, yet the way that words are distributed across language samples suggests that human languages do not satisfy this requirement.

Considering the second point discreteness, although writing conventions lead to some systematic agreements about what linguistic units are such that words are often thought of as standard discrete linguistic units, speech appears to be different. Human intuition on boundaries in speech diverge as exposure increases. When literate adults, nonliterate adults, and children are asked to divide a speech sequence into units, their intuitions on where any given sequence should be split into multiple units exhibit a systematic lack of agreement [6]; similar effects have been observed when people are asked to discriminate phonetic contrasts [7].

As for memorylessness, which supposes a distribution of events such that an event’s probability is independent of the way it is sampled, it has been shown that increased exposure to language leads to a decrease in the informativeness of high-frequency tokens relative to words that they co-occur with such that the informativity relationships between words appear to be unstable across cohorts [5]. For instance, the information that blue provides changes systematically as people successively hear about blue skies, blue eyes, and blue berries, etc. at different rates, an effect that increases nonlinearly with the number of blue covariates that speakers encounter.

To summarize these points, it is clear that adult expectations about events and their probabilities vary with experience. This in turn seems to suggest that the increasing divergence between individual speakers’ models will lead to an increase in communication problems between speakers. Nevertheless, sufficiently successful communication between speakers of different experience levels is not only possible but also relatively common. How?

Recent work by Ramscar [3] addresses these apparent communication problems from the perspective of discriminative learning and suggests that, unlike the predefined source codes in artificial communication, human communicative codes are subcategorized by systematic patterns of variation in the way words and arguments are employed. The empirical distributions discriminated by these patterns of variation both serve to minimize communicative uncertainty and to make unattested word forms predictable in context, thereby overcoming some of the problems that arise from the way that linguistic codes are sampled. In support of this argument, Ramscar presents evidence that the empirical distributions shaped by communicative contexts are geometric and suggests that the power laws that commonly characterize word token distributions are not in themselves functional but rather result from the aggregation of multiple functionally distinct communicative distributions [8]. Importantly, unlike power laws, the geometric distribution is sampling invariant and thus directly satisfies many of the constraints defined by information theory [9,10]. Perhaps even more importantly, geometric distributions also appear to maximize the likelihood that, independent of exposure, learning will lead to speakers acquiring similar models of distribution of communicative contrasts in context, thereby enabling a high degree of mutual predictability and helping to explain why human communicative codes actually work as well as they do.

A notable finding in this regard comes from an analysis of names (a universal feature of communicative codes that is almost universally ignored by grammatical theories) and in particular the distributions of English and Sinosphere first names [3,11]. This analysis shows that, historically, prior to the imposition of name laws that distorted their initial distributions, first names across a range of cultures had near identical geometric distributions. Names are a unique aspect of language in that their composition is highly regulated in virtually all modern nation states. Functionally, name sequences serve to discriminate between individuals, and thus, it follows that fixing distributions of name tokens by law in turn fixes the discriminatory power of those distributions. The 20th century is characterized by large global increases in population sizes; that is, the number of individuals that name distributions must serve to discriminate between has increased. In western cultures, this has had two consequences: first, the fixing of last names has caused the increase in information in the distribution of first names in direct proportion to increases in population [3,11]. Second, it has led to an increase in the diversity of regional first name distributions across very large states such as the United States. An interesting consequence of this is that, although the first name distribution in the US as a whole follows a power law, the distribution of names in the individual states still show close fits to the geometric, indicating that the shape of the former distribution may reflect the result of the aggregation of the latter [3,8].

These results suggest that, across space and time, discriminative codes somehow seem to respond to the various communicative pressures imposed by the environment in ways that sustain the sampling invariance that seems to be crucial to efficient, systematic communication, a point that name distributions in particular seem to underline in that individual contributions to the name pool appear, at least at first blush, to be somewhat random. These findings offer a new perspective on the apparent similarities and differences between communication in the human and information theoretical sense and raise some interesting questions in regard to speech. To what extent are speech codes shaped by the competing pressures of providing sufficient contrast to communicate the required distinctions while retaining a sufficiently stable structure to allow mutual predictability over the course of learning? Is the variance in the forms people actually articulate a consequence of the uncertainty in the structure of the immediate context in which they are learned and used, and does this variance have a communicative function?

The following sections briefly review the theoretical background to the present analysis. Section 1.1 reviews some key findings about linguistic distributions that appear to support their communicative function. Section 1.2 describes some of the implications of these findings for speech, and finally, Section 1.3 lays out a set of explicit predictions derived from this theoretical analysis. These are then examined in the rest of the article.

### 1.1. Grammar as Context—Convention Shapes Learning, Learning Shapes Context

It seems clear that human communication codes are not shared in the predefined way that information theory supposes [3]. Natural languages are learned from exposure to specific, incomplete samples, and these can diverge considerably across cohorts. This in turn suggests that any communicative system operating on global word token probabilities will be inefficient and unsystematic because the bursty/uneven distributions of low-frequency tokens observable in large language samples indicate that a large portion of types will be either over- or underrepresented across the communicative contexts any individual speaker is exposed to. At the same time, the fact that regularities in human languages can be consistently captured and shared through linguistic abstractions at many different levels of description suggests that speech provides speakers (and learners) with probabilistic structures that are sufficiently stable to ensure that most important linguistic conventions will be learnable from samples all speakers are exposed to. For example, Blevins et al. [12] suggest that the existence of grammatical regularities in the distribution of inflectional forms serves to offset many of the problems that arise from the highly skewed distribution of communicative codes, since the neighborhood support provided by morphological distributions makes forms that are otherwise unlikely to be attested to many speakers inferable from a partial sample of a code.

The fact that pseudowords can be interpreted in context [13] (for example, *He drank the dord in one gulp.*) offers another illustration of this point. Here, the lexical context provides sufficient support for the inference that *dord* is likely a drink of some sort, regardless of whether it is familiar to the speaker or correlated to a real life experience. (In the former case, if *dord* were to occur more regularly and in correlation to an actual bottled or cupped substance in the world, it would become a part of the vocabulary, losing its non-word status.) These kinds of context effects appear to rely on the fact that, in the sequences *drink milk*, *drink water*, and *drink beer*, *drink* systematically correlates with words that in turn covary with the consumption of fluids, unlike *eat apple*, *eat banana*, and *eat chicken*.

Given the discriminative nature of learning, it follows that exposure to samples containing this kind of systematic covariance structure will lead to the extraction of clusters (subcategories) of items that are less discriminated from any other items that occur in the same covarying contexts than to unrelated items [3]. Further, there is an abundance of evidence that patterns of systematic covariance of this kind provide a great deal of information, not only at lexical level (where semantically similar words typically share covariance patterns) but also at a grammatical level [3]. For example, in English, different subcategories of verbs can be discriminated from the extent to which they share argument structures with other verbs. The way that verbs co-occur with their arguments appears to provide a level of systematic covariance that nouns appear to lack [14]. For instance, the following sentences would be considered grammatical:

1. John *murdered* Mary’s husband.
2. John *ate* Mary’s husband.
3. John *chewed* Mary’s husband.

However, the following sentence would not be considered grammatical:

4. John *ran* Mary’s husband. (*)

One reason for this difference is that *chew*, *eat*, and *murder* share a similar pattern of argument structures (covary systematically) in a way that *run* does not. In contrast, the kinds of grammatical context which predicts a noun (noun phrases) appears to allow any noun—the sentence is grammatical—irrespective of its likelihood (although, obviously, these will vary widely according to context).

5. John *ate*.
6. John *ate* cheese.
7. John *ate* cheese slowly with a toothbrush.

In other words, the systematic covariance of verbs in their argument structures appears to constrain their distribution in context far more than is the case for nouns.

8. Mary *loved*. (*)
9. Mary *loved* cheese.
10. Mary *loved* cheese slowly with a toothbrush. (*)

Accordingly, the distributional patterning of verbs thus appears to reduce uncertainty about not only the lexical properties of upcoming parts of a message but also its structure. In other words, because verbs take arguments, there ought to be less variance in their patterns of covariation and this ought to lead to less overall uncertainty in the context of verb arguments. Consistent with this, Seifart et al. [15] report that slower articulations and more disfluencies precede nouns than verbs across languages, raising further questions about the kind of information that is communicated by variational patterns in speech and, in particular, whether and to what degree, this kind of sublexical variance actually serves a communicative function.

In the next section, we review some evidence that suggests the interactions observed between uncertainty and articulatory variation may indeed be functional.

### 1.2. Sublexical Variation in Context

It is well established that isolated word snippets extracted from connected speech tend to be suprisingly unintelligible outside of their context. By contrast, when reduced variants are presented to speakers in context, they are able to identify the word without difficulty and to report hearing the full form [16]. Consistent with this, the effect of frequency on speech production has been shown inconsistent across registers, speakers, lexical classes, and utterance positions and there are opaque interactions between context, lexical class, and frequency range.

At first blush, these inconsistencies would appear to limit the scope of functional accounts of speech sound variance, and to date, the effects that are stable enough to be taken as evidence for functional theories are mostly to be found in preselected content words from the mid-frequency range, such that the effects reported rarely align with effects observed in the remaining (by token count, significantly larger) parts of the distribution.

For example, while function words, high frequency discourse markers, and words at utterance boundaries account for the largest portion of variance in speech, their exclusion from the analysis of speech sound variance is such a common practice that it might be considered a de facto standard [17]. Against this background, it is noteworthy that Bell et al. [18] report a divergence in the extent to which the articulation of function and content words across frequency ranges is affected by both frequency and the conditional probability of the collocates. While duration in content words is well predicted by the information provided by the following word but not the preceding word, the effect decreases as the frequency increases and shows a reverse pattern in function words. Similarly, van Son and Pols [19] report a reversal in the correlation between reduction and segmental information in low-information segments and segments at utterance boundaries. The effect of information content is reported to be limited by a *hard floor* in high-frequency segments; that is, most frequent segments fail to support the hypothesis. Standardizing the exclusion of misfits is controversial, especially given that they outnumber the tokens which are typically taken to confirm a hypothesis and account for the largest part of variance in speech [20,21].

The seemingly random and noisy variance in the speech signal appears systematically correlated with uncertainty about the upcoming part of the message. As an example, vowel duration in low- and mid-frequency content words is correlated to the information provided by the upcoming word [18]. Words in less predictable grammatical contexts are on average longer and more disfluent [22]. These fluctuations in duration and sequence structure have been shown to inform listeners’ responses. For instance, the duration of common segments in word stems differ between singular and plural forms [23]. Speakers appear to use acoustic differences in word stem as a cue to grammatical context (plural suffix), and incongruence between segmental and durational cues lead to delayed responses in both grammatical number and lexical decision tasks [24]. Similar effects occur at many other levels of description; for example, disfluent instructions (*the ... uhm ... camel*) lead to more fixations to objects not predicted by the discourse context [25] and facilitate prediction of unfamiliar objects [26].

The occurrence of silent and filled pauses has been shown to contribute to the perception of fluency [27] and intelligibility [28] as well as improved recall [29]. Importantly however, neither artificially slowed-down speech samples nor samples modified by insertion of pauses are then perceived to be more fluent or intelligible, and indeed, in both cases, these manipulations have been shown to result in impaired performance [30]. Accordingly, the fact that listeners easily interpret reduced sequences from context and reject speech artificially altered to mimic completeness and fluency indicates that hearers are highly sensitive to violations of their expectations about how natural speech should sound and not that they have a preference for completeness and slow and extreme articulation. However, despite the evidence that sublexical variation shapes speaker expectations about the upcoming content, its contribution to successful communication as an informative part of the signal has remained relatively unexplored to date.

However, it is clear that any quantification of the communicative contributions of sublexical variations in context will depend on a consistent definition of context. That is, in order to address the extent to which the quality of articulation and the observed variance in the signal interact with the remaining uncertainty about the message in general terms, it is necessary to first formalize a consistent subset of higher-level abstractions that systematically covary in the degree to which they contribute to uncertainty reduction. The contrast between these subsets can then allow these effects to be analyzed independent of the specific context of any given utterance.

### 1.3. The Present Study

In comparison to written language, speech often appears to be messy. Instead of the well-formed word sequences that characterize text, spontaneous speech sequences are typically interrupted by silent and filled pauses, left unfinished, depart from word-order conventions, frequently miss word segments or whole words, and rely on clarifying feedback which tends to be short and grammatically incomplete. In consequence, the token distributions that underlie the information structure of written and spoken language differ substantially.

For instance, nouns are less lexically diverse in spoken English then in writing (based on measures derived from the Corpus of Contemporary American English (COCA)), whereas English adjectives tend to be more lexically diverse in speech. While reading and writing are self-paced, speech gives both speakers and hearers less control over timing. This suggests that the moment-to-moment uncertainty experienced in communication may differ in speech as compared to written language, and it may be that more effort is invested in uncertainty reduction in spoken than in written language. From this perspective, the increase in the lexical variety in prenominal adjectives, which in English reduce uncertainty about upcoming nouns [31], might be functional in that it may help manage the extra uncertainty in spoken communication. This raises the question of the degree to which these and other variational changes in spoken English are indeed informative and systematic.

These considerations also suggest that the results of previous analyses of the distributional structure of lexical variety in communicative contexts conducted on text corpora can only offer indirect support when it comes to answering questions about the communicative properties of speech. To address this shortcoming, we conducted a corpus analysis of conversational English [32] to explore the extent to which the distribution and the underlying structure of the grammatical context in which words are embedded interacts with speech signal variation observed across lexical categories. The goal of this analysis was to explore the structural properties of grammatical regularities in speech and their effect on the distributions of the lexical and sublexical contrasts that they discriminate between.

The analysis was conducted in two stages. Part one, presented in Section 3, addresses the distribution of grammatical and lexical contrast in speech and aims to answer the following questions:

- Are distributions of grammatical regularities in speech sampling invariant?
- How do recurrence patterns of grammatical categories and speech sequences inform learning?
- Are distributions of subcategorization frames and types they distinguish between geometric?

Part two of our analysis, presented in Section 4, assesses the concrete consequences of the sublexical variation observed in the speech signal and relates these to the results presented in Section 3, addressing the following questions:

- Are the inconsistent effects of frequency on speech sound variation across categories correlated with structural and distributional aspects of the grammatical and lexical contexts they populate?
- Finally and perhaps most importantly, is the resulting sublexical variance systematic?

## 2. Materials and Methods

### 2.1. Data

The Buckeye Corpus [32] contains phonetically transcribed speech from informal interviews with 40 speakers from Columbus, Ohio. The 286,982 words are annotated with a set of 41 standard aligner phone labels expanded by a set of markers for manner of articulation (nazalization, flaps, glottal stops, and retroflex vocalization). The corpus version we used was extended by Dilts [33] with measures of segmental deletion; dictionary form alignment; and deviation rate normalized by word length, speech rate, and backward and forward conditional probabilities of word ngrams. For the analysis reported here, we excluded from the corpus 8426 words with missing or incomplete duration variables. The data set and the code for the analysis can be found at https://osf.io/bqepj/.

In each of the 1-h interviews, the 40 speakers (who are balanced by age and gender) showed an enormous amount of variability (as assessed by phonetic transcription) in the speech signal. Overall, only 40% of the words are produced in their citation form. Only 38% of word types tend to appear in their non-citation variants more often and the propensity of individual speakers to pronounce word types in their citation form varies widely (between 36% word types and 67% word types). The word *that* appears in 313 variants, including *d ah tq*, *m ah t*, *z eh tq*, and *ng ah*.

We extracted for each citation form and parts-of-speech combination the number of variants observed in the corpus by citation form. The relative frequency counts for each form by parts-of-speech label were taken from the spoken part of COCA, an 80 million token subcorpus of Contemporary American English from transcripts of unscripted conversation on TV and radio programs.

### 2.2. Probability Distributions Analysis

Plotting a frequency distribution on a log-log plane, with log frequency on the y axis and log of rank order on the x axis, is a common method in the analysis of probabilistic structure. A linear plot indicates that the data conforms to Zipf’s law because Zipf’s law assumes an exponential increase in the time rate (rank). That is, a linear plot confirms a power law, while distributions we observe here and other variants of aggregate distributions (e.g., Zipf-Mandelbrot) are reported as an anomaly. The latter usually entails the introduction of additional parameters to fit the distribution back to power law.

Because linguists have so far only searched for power laws, the distributions we observe here are, when found, reported as an exception [34]. Ramscar [3] argues that empirical linguistic distributions ought not to be expected to follow power laws. Rather, because learning and mutual predictability require a regular distribution of events over time, human communicative codes ought to be expected to have distributions that retain their structures over time. Accordingly, following Ramscar [3], we employed log-linear plots in these analyses. That is, the linear decrease in probability over discrete time defines a time invariant communicative distribution while the exponential decrease in probability does not. To asses the extent to which the method captures this property, we apply it to a set of subsamples drawn from the original data.

**Figure 1:**
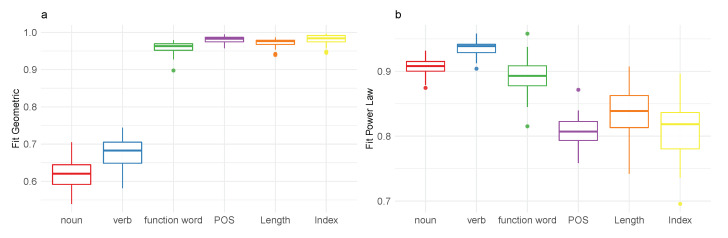

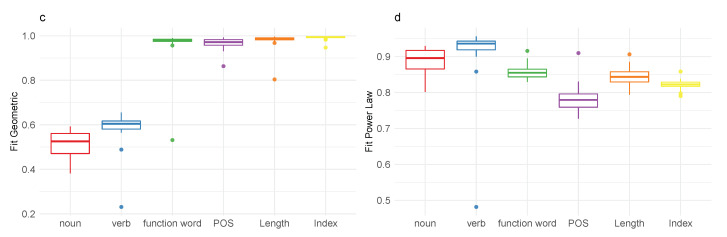
Boxplots of fits to geometric distribution (**a**,**c**) and power law distribution (**b**,**d**) for categories analyzed in Section 3.1 and Section 3.2 for the first 2500 words by 40 speakers (**a**,**b**) for 40 random samples ranging in sizes between 652–19,363 (**c**,**d**).

Figure 1a,b shows results from a simulation study capturing fits of the analyzed categories to a geometric distribution and a power-law distribution, respectively, over the first 2500 words from each of the 40 speaker subsamples. The two bottom row panels show the fits to geometric (Figure 1c) and power law (Figure 1d) across 40 random subsamples varying in size between 652 and 19,363 tokens. As we can see in Figure 1, fits to power law vary with sample size and source across all categories. In contrast, fits to geometric remain relatively stable in empirical distributions independent of sample source and size. This is not the case for aggregate distributions. Accordingly, this method appears to capture the critical property of communicative distributions addressed in this paper.

### 2.3. Statistical Analysis

The results presented in Section 4.1 were analyzed with a generalized additive mixed-effects model (GAMM) [35,36], working with the *mgcv* package for *R*. GAMMs are used for the analysis of complex, often nonlinear patterns involving the interaction of two or more numeric and factorial predictors. Instead of using polynomial functions, GAMMs introduce smoothing splines. A smoothing spline with one predictor fits a curve over multiple basis functions. Smoothing splines with multiple predictors fit multidimensional surfaces. These features allow us to explore interactions between frequency range, context, and lexical category and to reduce the model complexity by identifying relevant predictors which eventually result in linear effects.

## 3. The Structure of Lexical and Grammatical Variety in Speech: A Corpus Analysis

### 3.1. Part-of-Speech Token Distributions

#### 3.1.1. Why Parts of Speech?

It is clear that many important regularities in human languages are consistently captured by high-level linguistic abstractions such as, for example, parts-of-speech categories, indicating that languages may be sufficiently structured to allow the discrimination of various functional parts of codes at various levels of abstraction. Ramscar [3] suggests that the probabilistic co-occurrence patterns of words and phrases serve to discriminate subcategories of signals (and hence codes) and that, as well as serving different communicative purposes, these subcategories form distributions that facilitate speaker alignment at various levels of analysis. This raises an obvious question: do the distributional properties of structural regularities in conversational speech actually support this hypothesis?

Parts-of-speech tags are often used to label the various categories that can be extracted from the abstract structure of languages. Different tag sets are used for languages which differ in structure, and the extent to which tags capture detail varies with the particular context in which tagging is employed. These tags are assigned automatically by statistical tools, typically assuming a Markov process, which employs regularities in word co-occurrence patterns over word sequences of varying sizes [37,38]. The fact that taggers achieve high levels of accuracy suggests in turn that high levels of systematicity must be present in distributional patterns. That is, the fact that structural properties of the training set will translate to novel and larger samples implies that the captured properties are sampling invariant. Previous work on text corpora implies that, in text at least, the empirical distributions discriminated by communicative contexts are geometric [3]. This raises a question: do the patterns that emerge during part-of-speech tagging also discriminate distributions with similar empirical properties?

Further, the finding that the probability of types that are subcategorized by these context decreases at a constant rate [3] suggests in turn that different empirical subcategories might serve similar communicative purposes at different levels of specificity. In English, message length in words has been shown to increase as the content of messages increases as a consequence of learning and specialization [39,40,41]. The apparent systematicity revealed by analyses of covariance patterns in text suggests that communicative codes may be adapted to support the transmission of an unbounded set of messages at multiple levels of description, including length. That is, in speech at least, the considerations reviewed above would seem to suggest that word sequence length (at least in English) may be related to the relative probability of the message with respect to all messages all speakers might want to communicate. This raises a further question: is the distribution of n-grams in speech geometric?

To answer this, we analyzed the distributions of part-of-speech labels, utterance length, and utterance position in the Buckeye Corpus of conversational English [32].

#### 3.1.2. Results

**Figure 2:**
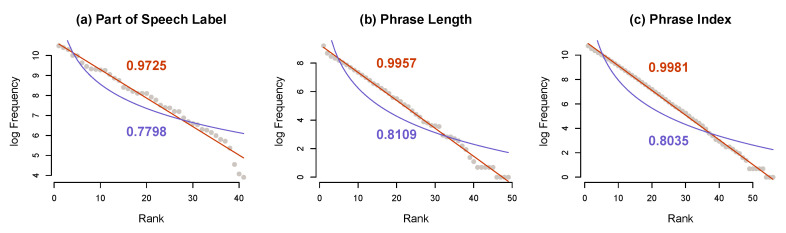
The frequency distributions for the part of speech label (**a**), utterance length (**b**) and utterance position (**c**) categories in the Buckeye Corpus [32]: Grey points show the observed distribution, with fits to a power law distribution (blue line) and a geometric distribution (red line). All three distributions show a close fit to a geometric distribution.

Figure 2a–c shows frequency rank distribution plots of log counts for part-of-speech labels, phrase lengths, and the phrase positions, respectively. The blue line indicates the best fit to log-log scale (power law), while the red line shows the best fit to geometric (geometric is linear; the probability decreases at a constant rate). As we can see, the empirical distribution (represented by the grey points) of part-of-speech labels, utterance length, and utterance position, with $R^{2}$ of 0.9725, 0.9957, and 0.9981, respectively, show a close fit to geometric, whereas fits to power law are 0.7798, 0.8109, and 0.8035, respectively.

These results thus suggest that sampling from the functional distributions that can be discriminated by context at this level may indeed result in probability estimates that are similar across speakers, irrespective of discourse context and length.

In addition, they provide some evidence to support the suggestion that hearing a one-word utterance such as *yes*, *okay*, *correct*, or *exactly* or a longer utterance such as *um sort of let them make their own decision when they got older what they wanted to do* is sufficiently stable irrespective of size, again indicating that the distribution of communicative types at different levels of description in conversational speech may be systematic.

#### 3.1.3. Discussion

Our results show that grammatical subcategories captured by part-of-speech tags have distributions that are likely to lead to an alignment in the probabilistic expectations of speakers regardless of any differences in their exposure to these distributions. They also provide further support for the suggestion that, unlike aggregate word token distributions (which have power law distributions [42]), the empirical distributions that are discriminated by communicative context are geometric [3].

The abstract model of communication defined by Shannon [9] is at heart a deductive process of uncertainty reduction [3]. The model assumes that communicative codes will be distributed so as to ensure that every sequence produced has the same statistical properties. A consequence of this is that any mixture of code samples will have the same statistical properties as any other sample. By contrast, it would appear that, in speech at least, natural languages gradually reduce message uncertainty via a series of sequential subcategorization frames of increasing degree of specificity. Evidence for this suggestion is provided by the differences in type/token ratio of part-of-speech categories, which vary systematically. Further, the shape of the distribution of utterance lengths suggests that the expectations about the distribution of messages of different lengths that speakers learn will likely align, helping the overall system to deal flexibly with the ever-growing number of specific messages that humans are likely to wish to communicate.

In the introduction, we described how constraints on the structure of name sequences have lead to qualitatively different patterns of distribution in English and Sinosphere first names. Legal constraints on last names in English have lead to differentiation between (geometric) local first name distributions which, when aggregated over, fit power laws [3]. Thus, the differences in the extent to which word categories are subjected to grammatical and lexical constraints (Section 1.1) seem to predict differences in the productivity of lexical categories over time, leading to more aggregation in verbs. The analysis presented in the next section aims to explore whether the shape of word frequency distributions of different lexical categories reflect the differences in the way they are constrained by the grammar.

### 3.2. Covariance, Systematicity, and Subcategorization

#### 3.2.1. Token Distributions across Lexical Categories

As we noted above, high-level descriptions (e.g., parts-of-speech) clearly capture many abstract communicative properties such as animacy, agency and number in nouns or tense, and aspect or argument structure in verbs. However, it seems that the functionality of these categories is further subcategorized by patterns of co-occurrence which encode more specific distinctions between agents, objects, actions, and relationships. This implies that verb and noun frequency distributions are aggregates of functionally distinct subcategories. Consistent with this, Bentz et al. [43] show that aggregates over verbs and nouns are power law distributed while Ramscar [3] confirmed this finding and then showed that the subcategorical distributions of verb and nouns discriminated by communicative context are geometric.

Importantly, previous studies have shown that token distributions in closed class categories (function words and modal verbs) do not follow power laws [43,44]. These departures from the trend to power law in other categories are assumed to be related to the communicative function of high-frequency words. Linguistic theories typically assume that closed class tokens serve a qualitatively different modifying or grammatical function while open classes are considered to contain and transport meanings; that is, they provide lexical contrast.

These previous results thus predict that, when context is not used to subcategorize them, nouns and verbs in English will be distributed differently to function words. To explore these patterns of distribution, we analyzed the word token distributions of these separate parts of speech across the speech samples.

#### 3.2.2. Results

There are 44,722 noun and 45,159 verb tokens in the analyzed sample. With 5817 unique types, nouns are a far more lexically diverse category than verbs with 2574 types. By contrast, the 116,960 function word tokens are represented by 144 unique types.

**Figure 3:**
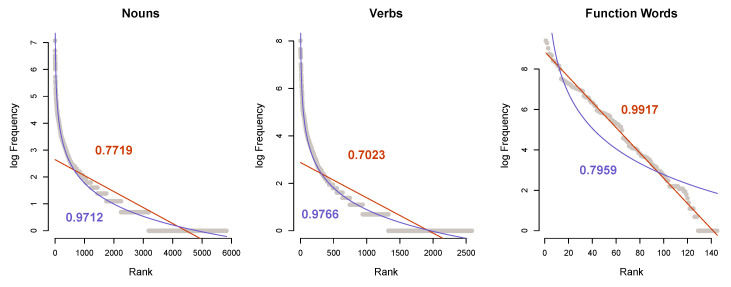
Word frequency distributions of nouns, verbs, and function words in the Buckeye Corpus [32] show that the substantially smaller (compared to nouns) set of verbs has a closer fit to power law distribution, indicating more aggregation. The shape of the distribution in function words suggests that function words form a natural empirical distribution.

Figure 3 shows the token distribution of the three largest grammatical categories. We can see that both verbs and nouns have a closer fit to power law compared to geometric distribution: $R_{pl}^{2}=0.976$ and $R_{geom}^{2}=0.701$ for verbs; $R_{pl}^{2}=0.971$ and $R_{geom}^{2}=0.772$ for nouns.

By contrast, the 144 unique function words ($n_{tokens}=11,696$) show an almost perfect fit to geometric $R_{pl}^{2}=0.796$, $R_{geom}^{2}=0.992$. A separate analysis shows a better fit to geometric over power law in distributions of determiners (n=16, $R_{geom}^{2}=0.953$, $R_{pl}^{2}=0.830$), pronouns (n=28, $R_{geom}^{2}=0.957$, $R_{pl}^{2}=0.741$), and prepositions/subordinating conjunctions (n=78, $R_{geom}^{2}=0.983$, $R_{pl}^{2}=0.863$). The aggregated set of function words, however, improves the fit to geometric.

#### 3.2.3. Discussion

When taken in conjunction with earlier findings [3,43,44], the distribution of function words we observed here supports the suggestion that they form a natural communicative distribution. This in turn suggests that, despite the fact that prepositions (in contrast to determiners and pronouns) distinguish between spatial and temporal relations, prepositions, determiners, and pronouns are part of the same functional subsystem and, at some level, serve the same communicative function.

By contrast, we find that the lexically more diverse categories fit power laws. As previously discussed, these distributions could be the product of aggregating over multiple communicative distributions serving distinct communicative functions. This suggestion is further supported by the observed distributions of verbs and nouns, which suggest that a smaller number of unique verb types appears across a larger number of distinct communicative contexts than is the case for nouns. This observation is supported by the fast growing head in the verb distributions, which appears to result from aggregating over high-frequency verbs, whereas the fast growing tail in the noun distributions appears to reflect the greater volume of low-frequency nouns.

In other words, the results imply that the differences observed between lexical categories do not necessarily warrant categorial distinctions. Rather, the observable differences appear to reflect the extent to which word co-occurrence clusters are shaped by the opposing communicative pressures of prediction and discrimination over the course of learning.

In other words, these results confirm the idea that lexical categories are not equally distributed across utterance positions. The next part of our analysis explores these relationships further.

### 3.3. Lexical Category, Word Order, and Recurrence Patterns

#### 3.3.1. What Makes a Lexical Category?

The distribution of function words suggests that function words will form the grammatical subcategory that is first discriminated systematically from the speech signal. As a consequence, it seems likely that, as both intuition and many linguistic theories would predict, function words provide a first contextual frame to aid in the learning of other grammatical and contextual categories. Once these basic contextual frames are learned, they will provide context, assisting in the learning of other words. The idea that context will provide information that aids learning in turn suggests lexical diversity will increase with utterance length.

Consistent with this suggestion, Genzel and Charniak [45] have shown that, although caching local probability estimates of a words’ occurrence in written samples (to account for the variance in recurrence patterns over time) stabilizes relative entropy over lexical sample size in nouns significantly, the effect is far smaller in verbs and absent in function words. In the light of the foregoing discussion, this might be taken to suggest that patterns of co-occurrence in verbs are less variant than those in nouns and that these patterns are still less variant (and may even be regular) in function words. These considerations suggest in turn that the different subcategories of words systematically reduce uncertainty in communication at different levels of abstraction. To explore whether the different communicative contributions of words from different lexical categories are quantifiable in speech signal, we analyzed the patterns of occurrence of nouns, verbs, and function words (the three largest categories by token count) over utterance length.

#### 3.3.2. Results

**Figure 4:**
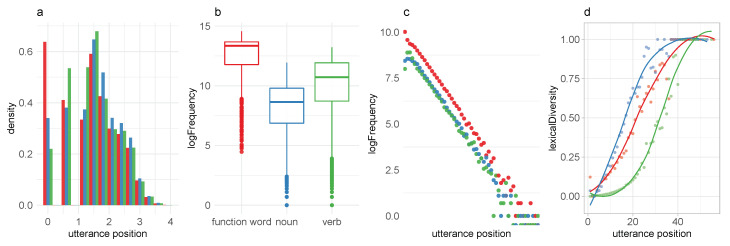
Distributional properties of the three largest (by token count) categories analyzed by utterance position and frequency range: We find that the overall probability of occurrence varies with type and utterance position (**a**), that frequency distributions of lexical classes are not evenly distributed across probability space (**b**), that part-of-speech token probability decreases linearly as a function of utterance position (**c**), and that lexical diversity increases nonlinearly as a function of utterance position (**d**).

The probability density of token occurrence over log normalized utterance length was analyzed by category. As can be seen in the left panel of Figure 4, while the larger parts of tokens in all three categories follow a normal distribution across utterance position (presumably as a consequence of utterance length), there is also evidence of distinct bursts of occurrence which align with the word order typology of English. That is, less specific pronouns are more likely in utterance initial positions, verbs are more likely in utterance medial positions, and nouns are more likely in utterance final positions.

The extent to which lexical categories are represented across the probability space (Figure 4b) is correlated to the average utterance position. We find 85% of all function word types in the top 50 tokens, which makes up 51% of the probability mass, and 93% of function word types in the top 100 words, which makes up 64.6% of the probability mass. In other words, function words are high-frequency words.

Further, we observe that, while token probability across lexical categories decreases linearly (Figure 4b) over utterance position, the increase in lexical diversity across all three categories is nonlinear. The right panel of Figure 4 shows smoothness of the normalized type/token ratio as a function of utterance position. We observe significant differences in the patterns of increase between the three lexical classes. The increase in the lexical variety of function words is limited to a small number of tokens in the latter positions of long utterances. The diversity in nouns increases earlier than in verbs.

**Figure 5:**
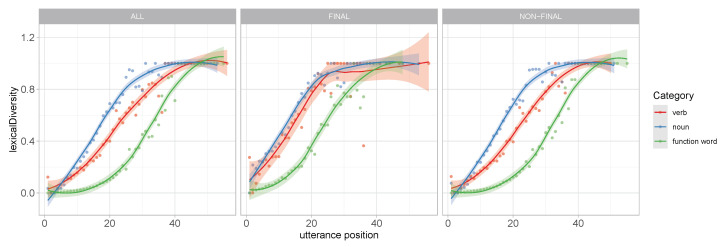
Increase in local lexical diversity (type/token ratio) across utterance position is not linear. The increase rates differ substantially between lexical classes. The differences in the increase rate between verbs and nouns in utterance final position are restricted to utterance initial tokens. The confidence interval in verbs is larger. The differences in the increase rate between verbs and nouns are constituted by the extent to which context affects lexical variety in nonfinal tokens.

Figure 5 shows that when words at utterance boundaries are excluded from the analysis, the normalized type/token ratio of nouns and verbs show similar increase patterns while the growth in function words remains unaffected. In contrast, the wide confidence interval in utterance final verbs indicates that the relationship between lexical diversity and utterance length (which can be taken to signify context) is less consistent in verbs than it is in nouns (and pronouns).

#### 3.3.3. Discussion

In sum, we observe significant differences in distributional properties between word categories. Word categories differ with respect to the frequency range they populate, the average utterance position, and lexical diversity. From the perspective of learning, this implies that the properties which distinguish word categories interact with the order in which they are learned while the order in which they are learned appears to be a consequence of the regularity with which they are represented across samples.

We suggest that the aggregation effects in token distributions across lexical classes is correlated to the degree in which category types are regularly distributed across language samples, reflecting the extent to which their communicative function is mediated by the contextual frames they appear within. Our analysis shows that lexical classes differ both in the average utterance position and in the rate at which lexical diversity increases as a function of utterance position and that the increase rate is inversely related to the average utterance position of the class.

In the next part of the analysis, we explore the extent to which the variety of abstract grammatical constructions in which words are embedded can serve to capture the differences in distributional structure and recurrence patterns that we observe across lexical categories.

### 3.4. Distribution of Grammatical Context

#### 3.4.1. How Do Different Parts of Speech Carry Out Their Communicative Function?

Words often occur in multiple grammatical contexts. The word *claim*, for instance, appears 5989 times in the spoken section of the Corpus of Contemporary American English [46]: 2719 times as a verb and 3270 times as a noun, 3016 times as noun singular, 1994 times as base form verb (1), and 1276 times as an infinitive (2), so that the three instances of *claim* in the three following examples serve distinct communicative functions which are not equally probable.

11. The girls *claim* to have seen the fairies.
12. You may be able to *claim* compensation.
13. The court found no evidence to support her *claim*.

The particular uses of *claim* that speakers intend to communicate will thus be determined by the lexical and grammatical context in which it is used. If one were to count the word *claim* as one type across all the contexts it occurs within without taking into consideration its lexical status, one would run the risk of aggregating over the multiple communicative functions it serves, and this problem will clearly increase as a word’s frequency increases.

To explore the extent to which lexical subcategories receive support from these kinds of contextual frames, we next analyzed the distributional properties of the frames that words are embedded within by lexical category.

#### 3.4.2. Results

In the first part of this analysis, we explored the distributions of grammatical context (defined as part-of-speech bigram) that words are embedded in. This was then followed up with an analysis of the word token distributions that these part-of-speech constructions distinguish between.

**Figure 6:**
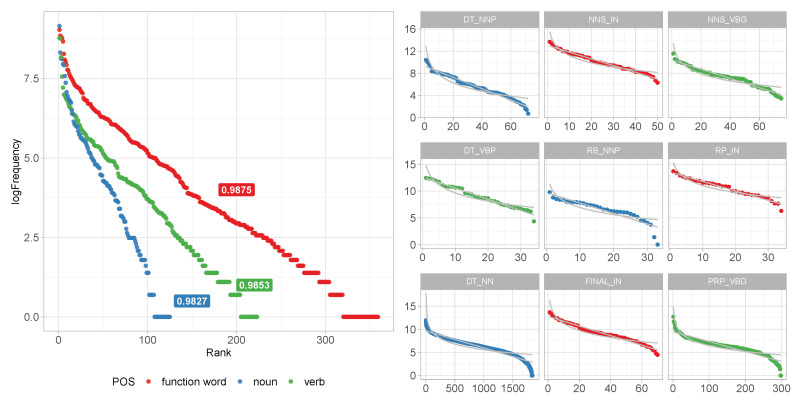
Distribution of contextual distinctions (part of speech bigrams) by lexical class: Nouns appear in a far smaller number of contextual frames; the size of the contextual frame is on average larger. The frequency distribution of verbs within the contextual frame is exponential. In the larger set of nouns, we see effects of aggregation in the low- and high-frequency tails.

The left panel of Figure 6 presents the log frequency rank plots of part-of-speech bigram distributions over the three analyzed lexical categories. It shows that all three distributions are geometric and that the slopes differ substantially. The slopes which reflect the extent to which words are subcategorized by grammatical context are inversely correlated to the rate at which lexical diversity increases as a function utterance position in Figure 5.

We observe a more diverse set of grammatical distinctions between categories of smaller frame size in verbs as compared to nouns. The distribution of the parts-of-speech bigram of the preceding word and the word itself is more diverse, with 124, 223, and 359 parts-of-speech constructions for 5869, 3124, and 144 unique word forms, respectively. The parts-of-speech context on average comprises 37 types of verbs (ranging from 1 to 460) and 119 types of nouns (ranging from 1 to 1862). In contrast, function word contexts on average host 2 distinct function words.

This suggests that the extent to which words are subcategorized by grammatical context is correlated with both lexical diversity and the average utterance position of a category. In consequence, the lexical distributions we find embedded in grammatical frames differ in size and structure. The center and the right panels of Figure 6 show the frequency distributions of the unique words found in two of the smaller subcategorization frames. The smaller (by unique type count) frames show a close fit to geometric irrespective of the word frequency range. In general, we observe more aggregation in noun frames. That is, the extent to which the subsamples extracted from grammatical subcategorization frames show the effects of aggregation appears to be independent of the frequency range of lexical contrast they distinguish between. Instead, aggregation appears correlated to the size of the subsample and, by implication, the extent to which lexical frames serve further subcategorization within the more abstract grammatical frames.

#### 3.4.3. Discussion

The distribution of grammatical constructions suggests that nouns which on average appear in a smaller number of more lexically diverse constructions will receive more support from lexical frames, resulting in less variance in the conditional probability between nouns and the words on which they are conditioned. That is, the more high-frequency nouns tend to appear in larger, high frequency contexts and thus tend to be further subcategorized by smaller lexical subcategorization frames. In contrast, the extent to which the variety of grammatical contexts serves to reduce uncertainty across a smaller (by type count) set of verbs will lead to more variance in the conditional probability between verbs and verb arguments.

In the next section, we explore the effects that the distinct patterns of covariance between high-frequency verbs and high-frequency nouns and their collocates have on the variety of articulated variants we find in the speech corpus.

## 4. From Information Structure to Speech

The results we have described so far suggest that the structure of speech serves to facilitate efficient message transmission over multiple nested levels of description. The distribution of lexical and grammatical contrasts indicates that information structure *depth* increases over message sequences, supporting gradual increases in the degree to which low-level sublexical contrasts contribute to resolving uncertainty about a message. Consistent with this, it has been shown experimentally that speech rates are perceived as being faster and target words as being longer when cognitive load is increased [47], a response pattern that suggests that speakers adapt their response to the relative uncertainty resulting from utterance context.

The notion that the timescale variance captured in speaker and listener performances reflects adaptation to uncertainty is further supported by evidence showing that the sublexical variation in speech sequences increases with sequence length, a phenomenon characterized by the strengthening of word initial consonants and the lengthening of final vowels. While both effects increase cumulatively as a function of utterance length [48], the interaction between lengthening and strengthening is weak, indicating that hyperarticulation and vowel space expansion are not equally affected by context. Moreover, while low-probability and word initial segments are more likely to be stressed and while segment deletion is more likely in high-frequency phonemes and in latter positions, the frequency effects actually observed in very frequent segments depart from this pattern. Also, the correlation between duration and extreme articulation, and duration and frequency declines as a function of utterance position [49].

The analyses presented in Section 3.4 indicate that average grammatical uncertainty peaks in words that are more likely to occur in utterance initial positions and that average lexical uncertainty peaks in categories which are more likely at utterance final positions. It has also been shown that slow-downs in articulation are associated with uncertainty and that uncertainty leads to an increase in articulatory variance. These effects have been observed both within [23] and across word boundaries [18] as a consequence of syntactic irregularities [22] and appear functional in lexical decision [24] and discourse [26]. Since our analyses show substantial differences across parts of speech in both the extent to which words are predicted by the previous context and the extent to which they serve to predict the upcoming part of the message across the frequency range, this seems to imply that the apparently inconsistent effects of frequency that have been previously observed are both predictable and systematic with respect to the structure of the grammatical context.

This in turn can be taken to suggest that sublexical variance follows as a consequence of an increase in lexical and grammatical variety in which words are embedded and that the variants we observe aim to increase the efficiency in transmission of informative contrast at multiple levels of description. In the next section, we conduct a statistical analysis of the effects of variation in the collocations of words on the number of distinct forms found in the speech corpus.

### 4.1. Effects of Frequency and Collocate Diversity on Variation

#### 4.1.1. The Distinct Effects of Collocate Diversity And Frequency

Wedel and colleagues have shown that the number of competing minimal pairs in lexical context predict likelihood of vowel merger [50] and voice onset time duration [17], suggesting that what drives speech contrast loss is the extent to which minimal pair competition is resolved in context. In line with this, Piantadosi et al. [51] observe that the relative probability of a word in a lexical context (defined as word sequences ranging between 2–4 words) is a far better predictor of word length than word frequency.

This raises questions: Does this hold for variance too? Is the diversity of collocate contexts across which a word appears a better predictor of the extent to which a type will vary across a speech sample than frequency?

The probability of a known word appearing in a previously unattested context increases with the average word count so that word frequency and collocate diversity are strongly correlated (r(9190)=0.70, p<0.0001). High-frequency words are more likely to be preceded by a larger number of different words and thus tend to appear across a larger number of communicative contexts that vary in size. By implication, there is more variance in the conditional probability between high-frequency words and their collocates. In contrast, words from the mid-frequency range will appear in a smaller number of distinct communicative contexts, leading to less variance in the conditional probability between mid-frequency words and their collocates. In line with this, an analysis by Arnon and Priva [52] shows that, in contrast to results reported by Bell et al. [18], duration in content words is affected by both word and multiword frequency as well as the transitional probability of both the preceding and following collocates when high- and low-frequency trigrams; sequences interrupted by pauses and word final sequences are excluded from the analysis. Finally, the increase in lexical diversity over utterance length (Section 3.3) suggests that low-frequency words tend to appear in a larger number of distinct message contexts, again leading to more variance in the conditional probabilities of low-frequency words at different positions within the sequence with respect to the likelihood of the message.

The discriminative nature of learning predicts that this variance will increase within-context competition over exposure time and that this will minimize the informativeness of contextual cues which predict a large number of lexical contrasts. This in turn predicts more sublexical variation in words that serves as cues to a larger number of collocates, reflecting the uncertainty of the relative context. Taken together, these factors predict distinct patterns of variance across frequency ranges.

#### 4.1.2. Results

To explore the nonlinear effects of frequency and collocate diversity on observed variance, we fitted generalized additive mixed models (GAMM) [35] using the *mgcv* package for R. In baseline model 1, we model the normalized number of observed corpus variants as a function of the smooth over log frequency. In baseline model 2, we model the number of variants as a function of a smooth over collocate diversity, the log normalized number of preceding words we observe in the corpus.

Model 1 counts show a strong, nonlinear effect of frequency (p<0.0001). It yields an $R^{2}$ of 0.435 and explains 43.5% of the deviance in the data (edf=5.05, AIC=19,852.04). Model 2 shows a strong, nonlinear effect of diversity of collocates in the preceding position (p<0.0001), explaining 74.6% of the variance in the data (edf=6.922, $R^{2}=0.746$, AIC=11,777.66).

We assessed the goodness of fit of both models by the Aikake Information Criterion (AIC). Model 2 improved the score by 8074.38. To contrast the contribution of both predictors, we modeled word variance as a function of smooth over log normalized word frequency and log normalized number of variants observed in the corpus in a combined model 3. Model 3 ($R^{2}=0.746$, AIC=11,548.74) reduced the AIC by 228. Both predictors are highly significant (p<0.0001).

**Figure 7:**
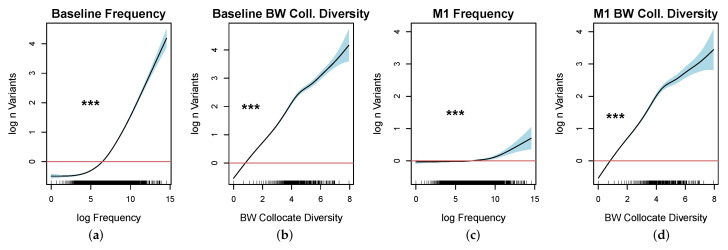
Baseline word variance model comparison: (**a**) the log normalized number of observed variants as a a function of smooth over log frequency (derived from the spoken part of COCA); (**b**) the log normalized number of observed variants as a function of collocate diversity, the log number of preceding words; and (**c**,**d**) Figure 7a,b in a combined model.

Interestingly, the plots show that the frequency effects predicted by the baseline model 1 and the combined model diverge substantially across frequency ranges (see Figure 7a,c), suggesting that the effect of frequency is largely overestimated in the low-frequency and mid-frequency ranges by the baseline model. It further appears that a large part of the frequency effect is confounded by the correlation between word frequency and the number of collocate contexts a word appears within. There remains, however, a strong effect of frequency observable in high-frequency words. The high-frequency part of the data behind the effect comprises 82 function words, 57 nouns, and 47 verbs, representing 69%, 1%, and 2% of unique types, respectively.

Word frequency appears to influence the extent to which a word varies in form only in high-frequency words and thus holds for type variation across lexical categories to the degree with which the category is represented in the high-frequency tail of the word distribution. We further observe a stronger correlation between collocate diversity and word frequency in function words (r(143)=0.882, p<0.0001), than in verbs (r(2549)=0.665, p<0.0001) and nouns (r(5626)=0.593, p<0.0001).

**Figure 8:**
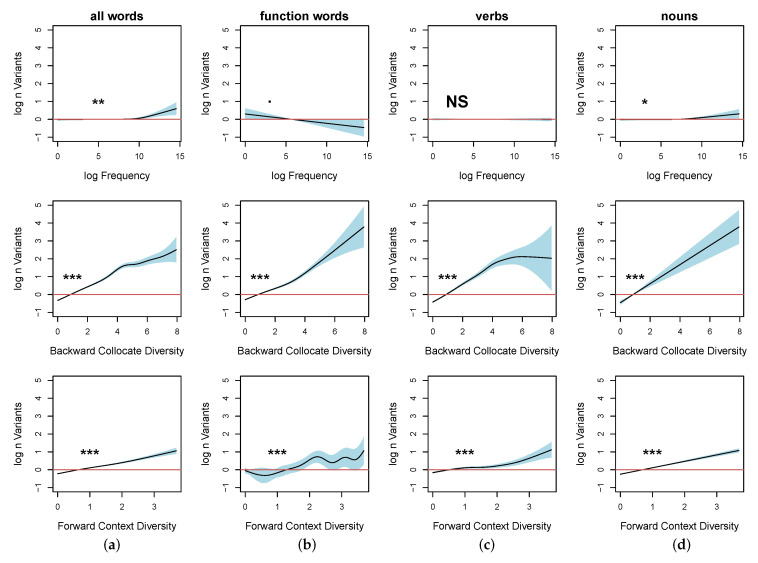
Log normalized number of observed variants as a function of smooth over log frequency (row 1) and adjacent token diversity (rows 2 and 3) for all words (**a**), function words (**b**), verbs (**c**), and nouns (**d**): when collocate diversity is taken into account, frequency effects on variation only hold in a minimal proportion of high-frequency nouns and appear to have no effect at all on verbs and function words.

Finally, we fitted a set of combined models, adding in the log number of distinct parts of speech following each word for all words (model 4) and adding in lexical category as a covariate factor (model 5). In model 4, we observe a fairly weak effect of frequency (p<0.006 (see Figure 8a), while the effects of the context predictors were strong. The AIC score is reduced by 531.

In model 5, the introduction of lexical category as a covariate further reduces the AIC score by 254 points and explains 76.8% ($R^{2}=0.766$) of deviance observed in the data. The effect of frequency is not significant in verbs (p<0.816) and function words (p<0.062) and is statistically significant but weak in nouns (p<0.018). Again, all contextual predictors are highly significant in function words, nouns, and verbs. The same pattern was observed for all of the other analyzed categories apart from the following exceptions: filled pauses and numbers show an effect of frequency (p<0.002); contractions are unaffected by the collocate diversity (p=0.21); and there is no interaction between modal verbs and the upcoming collocate context (p=0.1). Modals, numbers, and contractions comprise 0.008% of the analyzed data set. We observe differences in the effect of preceding collocate diversity between verbs and nouns in that the effect and the confidence interval both increase linearly in nouns while the effect levels off in high-frequency verbs, showing an increase in variance.

A closer examination of the data reveals that the relationship between word frequency and collocate diversity differ significantly across frequency ranges for verbs and nouns. Collocate diversity is much higher in high-frequency verbs and function words than it is in high-frequency nouns. Also, there is far more variance in the effect in high-frequency nouns.

#### 4.1.3. Discussion

The results of this analysis align with the finding that word counts outside of their communicative context contribute little when it comes to explaining variation in articulated forms. Rather, we observe that the largest part of this variance is explained by the diversity of the lexical contexts in which words appear. The remaining effects of frequency are limited to a relatively small number of high-frequency nouns and words from closed categories (numbers, contractions, and filled pauses).

These results are thus consistent with the differences we find in the distributional patterns of lexical categories in that, unlike high-frequency nouns, it would seem that high-frequency verbs are far less likely to be encountered outside of their argument frames (supporting the idea that verbs are encountered as arguments rather than lexical items per se).

Given that our results show that the variance in the observed forms is largely explained by the covariance in the collocate structure and that patterns of covariance are systematic, this finally leads us to the question of the systematicity in the sublexical variance: Is the distribution of the observed contrast geometric?

### 4.2. Distribution of Word Initial Contrast

#### 4.2.1. Why Word Initial Contrast?

Previous work on sublexical variation shows that the structure of speech sound sequences is such that the probability of speech segments at segment transitions is not independent [49]. Gating paradigm studies have shown that the informativeness of word medial contrast is mediated by the extent to which both the preceding sentence context and word initial phonetic contrasts have minimized uncertainty about the word [53,54]. Accordingly, the entropy in sublexical contrast peaks at word initial boundaries [49]. This suggests that word initial speech contrasts may serve a distinct communicative function in context.

An initial analysis of word initial phonetic label distributions over both observed and citation forms in the corpus revealed poor fits to both power law and exponential distributions, suggesting that the aggregated distribution of the phonetic labels observed in our corpus may result from mixing the underlying communicative distributions. To examine this, we used parts-of-speech classes to provide a simple, objective method for contextually disaggregating individual communicative distributions from the mixed distribution of phonetic labels in our corpus.

#### 4.2.2. Results

**Figure 9:**
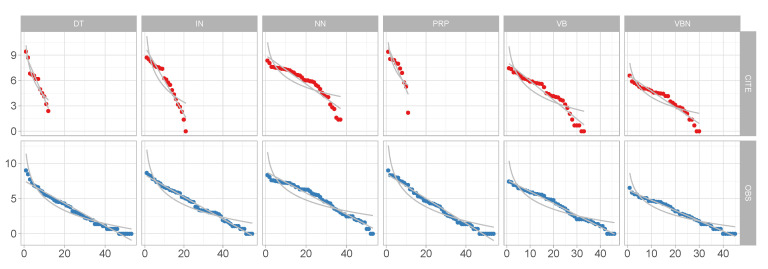
The distribution of word initial phonetic labels in 6 selected parts-of-speech categories: Row 1 shows the distribution presupposed by the dictionary forms, and row 2 shows the distribution of phonetic variants which were actually observed.

The frequency distributions of word initial phone labels were analyzed by parts-of-speech category considering the observed forms as the empirical distribution and the citation form as its model counterpart. Overall, both empirical and model distributions of phone labels show a better fit to geometric than to power-law distribution (Figure 9). However, while the fits to geometric in the model distribution show a larger departure from linearity and large differences in slope between different parts of speech, the observed phones across categories converge on nearly identical distributions with close fits to geometric (Table 1).

In both function and content words, the empirical distributions significantly improve the fit to a geometric. Importantly, despite substantial differences in the type/token ratio of the lexical classes analyzed, all of the categories have nearly identical empirical distributions with minimal differences in slopes. The exception is plural proper nouns where the data is extremely sparse (this category comprises a mere 50 tokens). Further, while we find that initial phones from several small categories (particles, modals, and filled pauses) have poor fits to either a geometric or a power law, in a similar vein, it is debatable whether these small sets of items constitute separate categories in terms of the covariate structures they populate.

Finally, we extracted time bins of initial phone duration centered by phone category to simulate an artificial set of discrete contrasts such that the simulation assumes a low-level subcategorization of phonetic contrast by duration. Again, across the parts-of-speech categories, the cumulative probability distributions of time bins show close fits to the geometric ($R^{2}>0.9662$) and poor fits to power law ($R^{2}<0.8333$).

#### 4.2.3. Discussion

Our analysis of word initial phonetic labels across different parts-of-speech categories confirms that they are geometrically distributed. The distribution of duration time bins is also geometric. These results thus suggest that what might appear to be random variance in the production of speech sounds may actually reflect a highly systematic distribution of sublexical contrasts.

While word initial variance is observable in all part of speech categories, we find that the extent to which tokens vary is closely correlated to uncertainty that is modulated by the underlying structure of the category. Importantly, despite large differences in the extent to which initial tokens deviate from the citation form, the probability distributions of tokens arising from this variance converge on nearly identical distributional properties across parts of speech.

Finally, we observe that the distribution of word initial phones assumed by the dictionary models show poor fits to geometric and power law, illustrating that, unlike the aggregate lexical contrasts, mixtures over closed sets of items similar in structure do not result in power laws. Instead, the distributions we observe are characterized by a fast growth in the mid-frequency range.

## 5. General Discussion

We analyzed distributions of the grammatical, lexical, and sublexical varieties in spontaneous conversational speech produced by 40 speakers of American English [32] to assess the effects of the statistical structure of speech on the sublexical variance observed in the signal. Our results show that distributions of regularities in co-occurrence patterns, the lexical contrasts they discriminate between, and the sublexical variety observed in the articulated forms result in distributions which are consistent with previous, similar analyses of written English that satisfy many of the communicative constraints described by information theory [3].

Accordingly, these results also provide further evidence that power law distributions seen in aggregate word frequency distributions are product of mixing functionally relevant distributions that are in themselves geometric [3,8].

The distributions in the analyzed sample suggest that, unlike the codes in artificial communication systems, human speech is a highly structured system of nested communicative distributions shaped by learning. In line with the predictions of learning theory, this suggests that speech variation at positions of high uncertainty is driven by the interaction of regular structures at multiple levels of description and that this variance serves to increases the efficiency of communication by increasing the amount of contrast in signals.

Taken together, our results indicate that the variance in the pronounced forms systematically structures the uncertainty discriminated by communicative contexts, supporting the suggestion that empirical distributions of phonetic contrasts in speech are components of a larger, highly structured communication system.

## Figures and Tables

**Table 1:** The distribution of word initial phonetic labels by part-of-speech category (Penn Treebank classification) from the Buckeye Corpus of conversational speech [32]: The first two columns contain slopes from the log frequency - rank model for observed and theoretical distributions, followed by the linear model fit to log frequency - rank ($R^{2}$, geometric), model fit to log frequency - log rank ($R^{2}$, power law) and the total number of assigned phonetic labels ($n_{phon}$). The model distribution represents the distribution of labels presupposed by the dictionary forms, while the empirical distribution shows phonetic contrast produced by the speakers.

Part of Speech	Slope		$R_{\mathrm{geom}}^{2}$		$R_{\mathrm{pl}}^{2}$		$n_{\mathrm{phon}}$	
	${}_{\mathrm{emp}}$	${}_{\mathrm{model}}$	${}_{\mathrm{emp}}$	${}_{\mathrm{model}}$	${}_{\mathrm{emp}}$	${}_{\mathrm{model}}$	${}_{\mathrm{emp}}$	${}_{\mathrm{model}}$
determiner	−0.16	−0.565	0.969	0.953	0.922	0.891	53	12
preposition	−0.157	−0.399	0.993	0.941	0.85	0.697	55	21
personal pronoun	−0.176	−0.554	0.984	0.802	0.87	0.593	54	11
noun, sg. or mass	−0.152	−0.171	0.975	0.895	0.726	0.621	53	37
proper noun, sg.	−0.14	−0.145	0.931	0.889	0.699	0.68	39	31
noun, plural	−0.167	−0.175	0.961	0.873	0.72	0.636	44	35
verb, base form	−0.168	−0.226	0.989	0.941	0.798	0.698	46	33
verb, past tense	−0.172	−0.244	0.972	0.944	0.784	0.768	44	31
verb, gerund/pres.part.	−0.168	−0.21	0.969	0.96	0.812	0.735	46	32
verb, past part.	−0.147	−0.193	0.991	0.934	0.828	0.716	45	30
verb, non-3rd pers.sg.pres.	−0.155	−0.249	0.991	0.977	0.777	0.751	51	31
verb, 3rd pers.sg.pres.	−0.157	−0.218	0.975	0.976	0.866	0.847	42	31
proper noun, pl.	−0.269	−0.342	0.847	0.908	0.938	0.958	12	10
function word	−0.15	−0.229	0.9731	0.8604	0.7317	0.6276	61	26
noun	−0.159	−0.199	0.9689	0.8713	0.7112	0.5797	54	40
verb	−0.164	−0.225	0.9856	0.9234	0.7406	0.6473	55	33
